# Large-Scale Phenotype-Based Antiepileptic Drug Screening in a Zebrafish Model of Dravet Syndrome^1^,^2^,^3^

**DOI:** 10.1523/ENEURO.0068-15.2015

**Published:** 2015-08-31

**Authors:** Matthew T. Dinday, Scott C. Baraban

**Affiliations:** 1 Department of Neurological Surgery, Epilepsy Research Laboratory, University of California San Francisco, San Francisco, California 94143; 2 Eli and Edythe Broad Center of Regeneration Medicine and Stem Cell Research, University of California San Francisco, San Francisco, California 94143

**Keywords:** antiepileptic, drug discovery, epilepsy, high throughput, pharmacology, zebrafish

## Abstract

Mutations in a voltage-gated sodium channel (*SCN1A*) result in Dravet Syndrome (DS), a catastrophic childhood epilepsy. Zebrafish with a mutation in *scn1Lab* recapitulate salient phenotypes associated with DS, including seizures, early fatality, and resistance to antiepileptic drugs. To discover new drug candidates for the treatment of DS, we screened a chemical library of ∼1000 compounds and identified 4 compounds that rescued the behavioral seizure component, including 1 compound (dimethadione) that suppressed associated electrographic seizure activity. Fenfluramine, but not huperzine A, also showed antiepileptic activity in our zebrafish assays. The effectiveness of compounds that block neuronal calcium current (dimethadione) or enhance serotonin signaling (fenfluramine) in our zebrafish model suggests that these may be important therapeutic targets in patients with DS. Over 150 compounds resulting in fatality were also identified. We conclude that the combination of behavioral and electrophysiological assays provide a convenient, sensitive, and rapid basis for phenotype-based drug screening in zebrafish mimicking a genetic form of epilepsy.

## Significance Statement

Dravet syndrome is a catastrophic childhood epilepsy that is resistant to available medications. Current animal models for this disease are not amenable to high-throughput drug screening. We used a zebrafish model for Dravet syndrome and screened >1000 compounds. We report the identification of compounds with the ability to suppress seizure behavior and electrographic seizure activity. This approach provides an example of precision medicine directed to pediatric epilepsy.

## Introduction

Dravet syndrome (DS) is a devastating genetic epileptic encephalopathy that has been linked to more than >300 *de novo* mutations in a neuronal voltage-gated sodium channel (*SCN*). Children with DS are at a higher risk for sudden unexplained death in epilepsy and episodes of uncontrolled status epilepticus (Dravet et al., 2005; Ceulemans et al., 2012). Delayed language development, disruption of autonomic function, and motor and cognitive impairment are also associated with this disease. Seizure management includes treatment with benzodiazepines, valproate, and/or stiripentol (Caraballo et al., 2005; Chiron and Dulac, 2011). Some reduction in seizure activity has been reported with the use of bromides and topiramate, or a ketogenic diet (Lotte et al., 2012; Wilmshurst et al., 2014; Dressler et al., 2015). Despite these options, available antiepileptic drugs (AEDs) do not achieve adequate seizure control in most DS patients (Dravet et al., 2005; Chiron and Dulac, 2011; Dressler et al., 2015), making the identification of new drugs a critical unmet need. High-throughput screening offers a powerful tool to identify new drug candidates for these patients. However, commonly available screening approaches rely on *in vitro* cell-based assays (Masimirembwa et al., 2001; Snowden and Green, 2008; Ko and Gelb, 2014) and do not recapitulate the complicated neural networks that generate seizures *in vivo*. Given the need for new treatments for children with DS, and the growing number of genetic epileptic encephalopathies that are medically intractable (Leppert, 1990; Epi4K Consortium, 2012; Ottman and Risch, 2012), we developed an alternative phenotype-based *in vivo* drug-screening strategy. While cell-based *in vitro* screening assays can efficiently identify compounds that bind specific targets, whole-organism-based screens are more likely to reliably predict therapeutic outcomes as they maintain the complex neural circuitry involved in the underlying disease process. Whole-organism screens do not require well validated targets to identify compounds that yield a desirable phenotypic outcome, but can be prohibitively costly and time consuming in mammals. As a simple vertebrate with significant genetic similarity to human, zebrafish are now recognized as an ideal cost-effective alternative to achieve rapid *in vivo* phenotype-based screening (Ali et al., 2011).

Using *scn1a* mutant zebrafish larvae with a gene homologous to human and spontaneously occurring seizures (Baraban et al., 2013), we screened, in a blinded manner, a repurposed library of ∼1000 compounds for drugs that inhibit unprovoked seizure events. We also screened two compounds (huperzine A and fenfluramine) that were discovered in rodent-based assays using acquired seizure protocols and that were recently suggested as potential treatments for DS (Boel and Casaer, 1996; Coleman et al., 2008; Ceulemans et al., 2012; Bialer et al., 2015). Only 20 compounds in the repurposed drug library reduced swim behavior to control levels. However, many of these compounds were toxic or were not confirmed on retesting, and only four compounds advanced to a second-stage *in vivo* electrophysiology assay. Of these compounds (cytarabine, dimethadione, theobromine, and norfloxacin) only dimethadione, a T-type calcium channel antagonist previously reported to have anticonvulsant activity (Lowson et al., 1990; Zhang et al., 1996), reduced ictal-like electrographic discharges seen in *scn1Lab* mutant larvae. This two-stage phenotype-based screening approach, using a genetic DS model with >75% genomic similarity to human, is a sensitive, rapid means to successfully identify compounds with antiepileptic activity.

## Materials and Methods

### Zebrafish

**Figure 1.**
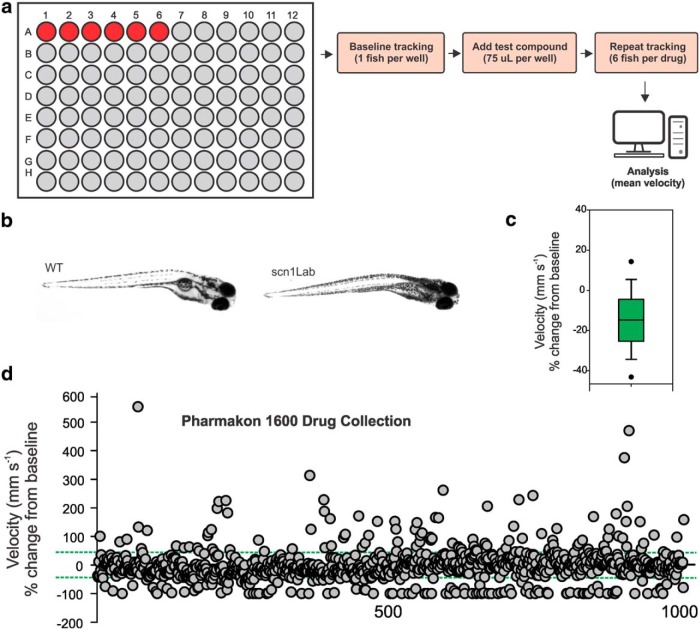
Locomotion assay to identify drugs that rescue the *scn1Lab* mutant epilepsy phenotype. ***a***, Schematic of the phenotype-based screening process. Chemical libraries can be coded and aliquoted in small volumes (75 µL) into individual wells containing one mutant fish. The 96-well microplate is arranged so that six fish are tested per drug; with one row of six fish maintained as an internal control (red circles) on each plate. ***b***, Representative images for WT and *scn1Lab* mutant zebrafish larvae at 5 dpf. Note the morphological similarity but darker pigmentation in mutant larvae. ***c***, Box plot of mean velocity (in millimeters per second) for two consecutive recordings of mutant larvae in embryo media. Experiments were performed by first placing the mutant larvae in embryo media and obtaining a baseline locomotion response; embryo media was then replaced with new embryo media (to mimic the procedure used for test compounds), and a second locomotion response was obtained. The percentage change in velocity from baseline (recording 1) versus experimental model (recording 2) is shown. In the box plot, the bottom and top of the box represent the 25th percentile and the 75th percentile, respectively. The line across the box represents the median value, and the vertical lines encompass the entire range of values. This plot represents normal changes in tracking activity in the absence of a drug challenge. ***d***, Plot of locomotor seizure behavior for *scn1Lab* mutants at 5 dpf for the 1012 compounds tested. Threshold for inhibition of seizure activity (positive hits) was set as a reduction in mean swim velocity of ≥44%; the threshold for a proconvulsant or hyperexcitable effect was set at an increase in the mean swim velocity of ≥44% (green dashed lines).

Zebrafish were maintained in a light- and temperature-controlled aquaculture facility under a standard 14:10 h light/dark photoperiod. Adult zebrafish were housed in 1.5 L tanks at a density of 5-12 fish per tank and fed twice per day (dry flake and/or flake supplemented with live brine shrimp). Water quality was continuously monitored: temperature, 28-30º C; pH 7.4-8.0; conductivity, 690-710 mS/cm. Zebrafish embryos were maintained in round Petri dishes (catalog #FB0875712, Fisher Scientific) in “embryo medium” consisting of 0.03% Instant Ocean (Aquarium Systems, Inc.) and 000002% methylene blue in reverse osmosis-distilled water. Larval zebrafish clutches were bred from wild-type (WT; TL strain) or *scn1Lab* (didy^s552^) heterozygous animals that had been backcrossed to TL wild-type for at least 10 generations. Homozygous mutants (*n* = 6544), which have widely dispersed melanosomes and appear visibly darker as early as 3 d postfertilization (dpf; Fig. 1*b*), or WT larvae (*n* = 71) were used in all experiments at 5 or 6 dpf. Embryos and larvae were raised in plastic petri dishes (90 mm diameter, 20 mm depth) and density was limited to ∼60 per dish. Larvae between 3 and 7 dpf lack discernible sex chromosomes. The care and maintenance protocols comply with requirements outlined in the *Guide for the Care and Use of Animals* (ebrary Inc., 2011) and were approved by the Institutional Animal Care and Use Committee (protocol #AN108659-01D).

### Seizure monitoring

Zebrafish larvae were placed individually into 1 well of a clear flat-bottomed 96-well microplate (catalog #260836, Fisher Scientific) containing embryo media. Microplates were placed inside an enclosed motion-tracking device and acclimated to the dark condition for 10-15 min at room temperature. Locomotion plots were obtained for one fish per well at a recording epoch of 10 min using a DanioVision system running EthoVision XT software (DanioVision, Noldus Information Technology); threshold detection settings to identify objects darker than the background were optimized for each experiment. Seizure scoring was performed using the following three-stage scale (Baraban et al., 2005): Stage 0, no or very little swim activity; Stage I, increased, brief bouts of swim activity; Stage II, rapid “whirlpool-like” circling swim behavior; and Stage III, paroxysmal whole-body clonus-like convulsions, and a brief loss of posture. WT fish are normally scored at Stage 0 or I. Plots were analyzed for distance traveled (in millimeters) and mean velocity (in millimeters per second). As reported previously (Winter et al., 2008; Baraban et al., 2013), velocity changes were a more sensitive assay of seizure behavior. For electrophysiology studies, zebrafish larvae were briefly paralyzed with α-bungarotoxin (1 mg/ml) and immobilized in 1.2% agarose; field recordings were obtained from forebrain structures. Epileptiform events were identified *post hoc* in Clampfit (Molecular Devices) and were defined as multispike or polyspike upward or downward membrane deflections greater than three times the baseline noise level and >500 ms in duration. During electrophysiology experiments zebrafish larvae were continuously monitored for the presence (or absence) of blood flow and heart beat by direct visualization on an Olympus BX51WI upright microscope equipped with a CCD camera and monitor.

### Drugs

Compounds for drug screening were purchased from MicroSource Discovery Systems, Inc. (PHARMAKON 1600) and were provided as 10 mm DMSO solutions (Table 1). Test compounds for locomotion or electrophysiology studies were dissolved in embryo media and were tested at an initial concentration of 100 µm, with a final DMSO concentration of <2%. In all drug library screen studies, compounds were coded and experiments were performed by investigators who were blind to the nature of the compound. Baseline recordings of seizure behavior were obtained from mutants bathed in embryo media, as described above; a second locomotion plot was then obtained following a solution change to a test compound and an equilibration period of 15–30 min. Criteria for a positive hit designation were as follows: (1) a decrease in mean velocity of ≥44% (e.g., a value based on the trial-to-trial variability measured in control tracking studies; Fig. 1*c*); and (2) a reduction to Stage 0 or Stage I seizure behavior in the locomotion plot for at least 50% of the test fish. Each test compound classified as a “positive hit” in the locomotion assay was confirmed, under direct visualization on a stereomicroscope, as the fish being alive based on movement in response to external stimulation and a visible heartbeat following a 60 min drug exposure. Toxicity (or mortality) was defined as no visible heartbeat or movement in response to external stimulation in at least 50% of the test fish. Hyperexcitability was defined as a compound causing a ≥44% increase in swim velocity and/or Stage III seizure activity in at least 50% of the test fish. Hits identified in the primary locomotion screen were selected from the PHARMAKON 1600 library and rescreened using the method described above. Select compound stocks that were successful in two primary locomotion assays, and were not classified as toxic in two independent clutches of zebrafish, were then purchased separately from Sigma-Aldrich for further testing. Drug concentrations between 0.5 and 1 mm were used for electrophysiology assays to account for more limited diffusion in agar-embedded larvae.

**Table 1.** List of compounds from the PHARMAKON 1600 library used in this screen.

ABACAVIR SULFATE
ABAMECTIN (avermectin B1a shown)
ACADESINE
ACARBOSE
ACEBUTOLOL HYDROCHLORIDE
ACECLIDINE
ACECLOFENAC
ACENOCOUMAROL
ACETAMINOPHEN
ACETOHYDROXAMIC ACID
ACETOPHENAZINE MALEATE
ACETRIAZOIC ACID
ACETYLCHOLINE CHLORIDE
ACETYLCYSTEINE
ACIPIMOX
ACONITINE
ACRIFLAVINIUM HYDROCHLORIDE
ACRISORCIN
ACTARIT
ACYCLOVIR
ADAPALENE
ADELMIDROL
ADENINE
ADENOSINE
ADENOSINE PHOSPHATE
ADIPHENINE HYDROCHLORIDE
AKLOMIDE
ALAPROCLATE
ALBENDAZOLE
ALBUTEROL (+/-)
ALENDRONATE SODIUM
ALEXIDINE HYDROCHLORIDE
ALLANTOIN
ALLOPURINOL
ALMOTRIPTAN
alpha-TOCHOPHEROL
alpha-TOCHOPHERYL ACETATE
ALPRAZOLAM
ALRESTATIN
ALTHIAZIDE
ALTRETAMINE
ALVERINE CITRATE
AMANTADINE HYDROCHLORIDE
AMCINONIDE
AMIFOSTINE
AMIKACIN SULFATE
AMILORIDE HYDROCHLORIDE
AMINACRINE
AMINOCAPROIC ACID
AMINOGLUTETHIMIDE
AMINOHIPPURIC ACID
AMINOLEVULINIC ACID HYDROCHLORIDE
AMINOSALICYLATE SODIUM
AMITRIPTYLINE HYDROCHLORIDE
AMLEXANOX
AMLODIPINE BESYLATE
AMODIAQUINE DIHYDROCHLORIDE
AMOROLFINE HYDROCHLORIDE
AMOXICILLIN
AMPHOTERICIN B
AMPICILLIN SODIUM
AMPROLIUM
AMSACRINE
ANASTROZOLE
ANCITABINE HYDROCHLORIDE
ANETHOLE
ANIRACETAM
ANISINDIONE
ANTAZOLINE PHOSPHATE
ANTHRALIN
ANTIPYRINE
APOMORPHINE HYDROCHLORIDE
APRAMYCIN
ARGININE HYDROCHLORIDE
ARMODAFINIL
ARTEMETHER
ARTEMOTIL
ARTESUNATE
ASCORBIC ACID
ASPIRIN
ATENOLOL
ATORVASTATIN CALCIUM
ATOVAQUONE
ATROPINE SULFATE
AUROTHIOGLUCOSE
AVOBENZONE
AZACITIDINE
AZASERINE
AZATADINE MALEATE
AZATHIOPRINE
AZELAIC ACID
AZITHROMYCIN
AZLOCILLIN SODIUM
AZTREONAM
BACAMPICILLIN HYDROCHLORIDE
BACITRACIN
BACLOFEN
BALSALAZIDE DISODIUM
BECLOMETHASONE DIPROPIONATE
BEKANAMYCIN SULFATE
BEMOTRIZINOL
BENAZEPRIL HYDROCHLORIDE
BENDROFLUMETHIAZIDE
BENORILATE
BENSERAZIDE HYDROCHLORIDE
BENZALKONIUM CHLORIDE
BENZETHONIUM CHLORIDE
BENZOCAINE
BENZOIC ACID
BENZONATATE
BENZOYL PEROXIDE
BENZTHIAZIDE
BENZYL ALCOHOL
BENZYL BENZOATE
BEPRIDIL HYDROCHLORIDE
BERGAPTEN
beta-CAROTENE
BETAHISTINE HYDROCHLORIDE
BETAINE HYDROCHLORIDE
BETAMETHASONE
BETAMETHASONE 17,21-DIPROPIONATE
BETAMETHASONE VALERATE
BETAMIPRON
beta-NAPHTHOL
BETAZOLE HYDROCHLORIDE
BETHANECHOL CHLORIDE
BEZAFIBRATE
BICALUTAMIDE
BIOTIN
BISACODYL
BISOCTRIZOLE
BISORCIC
BITHIONATE SODIUM
BLEOMYCIN (bleomycin B2 shown)
BRETYLIUM TOSYLATE
BRINZOLAMIDE
BROMHEXINE HYDROCHLORIDE
BROMOCRIPTINE MESYLATE
BROMPHENIRAMINE MALEATE
BROXYQUINOLINE
BUDESONIDE
BUMETANIDE
BUPIVACAINE HYDROCHLORIDE
BUPROPION
BUSULFAN
BUTACAINE
BUTAMBEN
BUTOCONAZOLE
CAFFEINE
CAMPHOR (1R)
CANDESARTAN
CANDESARTAN CILEXTIL
CANDICIDIN
CANRENOIC ACID, POTASSIUM SALT
CANRENONE
CAPECITABINE
CAPREOMYCIN SULFATE
CAPSAICIN
CAPTAMINE
CAPTOPRIL
CARBACHOL
CARBENICILLIN DISODIUM
CARBENOXOLONE SODIUM
CARBETAPENTANE CITRATE
CARBIDOPA
CARBINOXAMINE MALEATE
CARBOPLATIN
CARISOPRODOL
CARMUSTINE
CARNITINE (dl) HYDROCHLORIDE
CARPROFEN
CARVEDILOL
CEFACLOR
CEFADROXIL
CEFAMANDOLE NAFATE
CEFAMANDOLE SODIUM
CEFAZOLIN SODIUM
CEFDINIR
CEFEPIME HYDROCHLORIDE
CEFMENOXIME HYDROCHLORIDE
CEFMETAZOLE SODIUM
CEFOPERAZONE
CEFORANIDE
CEFOTAXIME SODIUM
CEFOTETAN
CEFOXITIN SODIUM
CEFPIRAMIDE
CEFSULODIN SODIUM
CEFTIBUTEN
CEFTIOFUR HYDROCHLORIDE
CEFTRIAXONE SODIUM TRIHYDRATE
CEFUROXIME AXETIL
CEFUROXIME SODIUM
CELECOXIB
CEPHALEXIN
CEPHALOTHIN SODIUM
CEPHAPIRIN SODIUM
CEPHRADINE
CETYLPYRIDINIUM CHLORIDE
CHENODIOL
CHLORAMBUCIL
CHLORAMPHENICOL
CHLORAMPHENICOL HEMISUCCINATE
CHLORAMPHENICOL PALMITATE
CHLORCYCLIZINE HYDROCHLORIDE
CHLORHEXIDINE
CHLOROCRESOL
CHLOROGUANIDE HYDROCHLORIDE
CHLOROQUINE DIPHOSPHATE
CHLOROTHIAZIDE
CHLOROXINE
CHLOROXYLENOL
CHLORPHENIRAMINE (S) MALEATE
CHLORPROMAZINE
CHLORPROPAMIDE
CHLORPROTHIXENE HYDROCHLORIDE
CHLORTETRACYCLINE HYDROCHLORIDE
CHLORTHALIDONE
CHLORZOXAZONE
CHOLECALCIFEROL
CHOLESTEROL
CHOLINE CHLORIDE
CICLOPIROX OLAMINE
CILOSTAZOL
CIMETIDINE
CINCHOPHEN
CINNARAZINE
CINOXACIN
CINTRIAMIDE
CIPROFIBRATE
CIPROFLOXACIN
CISPLATIN
CITALOPRAM HYDROBROMIDE
CITICOLINE
CLARITHROMYCIN
CLAVULANATE LITHIUM
CLEMASTINE
CLIDINIUM BROMIDE
CLINAFOXACIN HYDROCHLORIDE
CLINDAMYCIN HYDROCHLORIDE
CLIOQUINOL
CLOBETASOL PROPIONATE
CLOFARABINE
CLOFIBRATE
CLOMIPHENE CITRATE
CLONIDINE HYDROCHLORIDE
CLOPIDOGREL SULFATE
CLORSULON
CLOSANTEL
CLOTRIMAZOLE
CLOXACILLIN SODIUM
CLOXYQUIN
CLOZAPINE
COENZYME B12
COLCHICINE
COLFORSIN
COLISTIMETHATE SODIUM
CORTISONE ACETATE
COTININE
CRESOL
CROMOLYN SODIUM
CRYOFLURANE
CYCLAMIC ACID
CYCLIZINE
CYCLOBENZAPRINE HYDROCHLORIDE
CYCLOHEXIMIDE
CYCLOPENTOLATE HYDROCHLORIDE
CYCLOPHOSPHAMIDE HYDRATE
CYCLOSERINE (D)
CYCLOSPORINE
CYCLOTHIAZIDE
CYPERMETHRIN
CYPROTERONE ACETATE
CYSTEAMINE HYDROCHLORIDE
CYTARABINE
DACARBAZINE
DACTINOMYCIN
DANAZOL
DANTHRON
DANTROLENE SODIUM
DAPSONE
DAPTOMYCIN
DASATINIB
DAUNORUBICIN
DECIMEMIDE
DEFEROXAMINE MESYLATE
DEFLAZACORT
DEHYDROACETIC ACID
DEHYDROCHOLIC ACID
DEMECLOCYCLINE HYDROCHLORIDE
DERACOXIB
DESIPRAMINE HYDROCHLORIDE
DESOXYCORTICOSTERONE ACETATE
DESVENLAFAXINE SUCCINATE
DEXAMETHASONE
DEXAMETHASONE ACETATE
DEXAMETHASONE SODIUM PHOSPHATE
DEXIBUPROFEN
DEXLANSOPRAZOLE
DEXPROPRANOLOL HYDROCHLORIDE
DEXTROMETHORPHAN HYDROBROMIDE
DIAVERIDINE
DIBENZOTHIOPHENE
DIBUCAINE HYDROCHLORIDE
DICHLOROPHENE
DICHLORVOS
DICLAZURIL
DICLOFENAC SODIUM
DICLOXACILLIN SODIUM
DICUMAROL
DICYCLOMINE HYDROCHLORIDE
DIENESTROL
DIETHYLCARBAMAZINE CITRATE
DIETHYLSTILBESTROL
DIFLOXACIN HYDROCHLORIDE
DIFLUNISAL
DIGITOXIN
DIGOXIN
DIHYDROERGOTAMINE MESYLATE
DIHYDROSTREPTOMYCIN SULFATE
DILAZEP DIHYDROCHLORIDE
DIMENHYDRINATE
DIMERCAPROL
DIMETHADIONE
DIOXYBENZONE
DIPHENHYDRAMINE HYDROCHLORIDE
DIPHENYLPYRALINE HYDROCHLORIDE
DIPYRIDAMOLE
DIPYRONE
DIRITHROMYCIN
DISOPYRAMIDE PHOSPHATE
DISULFIRAM
DOBUTAMINE HYDROCHLORIDE
DOCETAXEL
DONEPEZIL HYDROCHLORIDE
DOPAMINE HYDROCHLORIDE
DOXEPIN HYDROCHLORIDE
DOXOFYLLINE
DOXYCYCLINE HYDROCHLORIDE
DOXYLAMINE SUCCINATE
DROFENINE HYDROCHLORIDE
DROPERIDOL
DROSPIRENONE
DYCLONINE HYDROCHLORIDE
DYPHYLLINE
ECAMSULE TRIETHANOLAMINE
ECONAZOLE NITRATE
EDETATE DISODIUM
EDITOL
EDOXUDINE
EMETINE
ENALAPRIL MALEATE
ENALAPRILAT
ENOXACIN
ENROFLOXACIN
ENTACAPONE
EPHEDRINE (1R,2S) HYDROCHLORIDE
EPINEPHRINE BITARTRATE
EPRINOMECTIN
ERGOCALCIFEROL
ERGONOVINE MALEATE
ERYTHROMYCIN
ERYTHROMYCIN ESTOLATE
ERYTHROMYCIN ETHYLSUCCINATE
ESCITALOPRAM OXALATE
ESOMEPRAZOLE POTASSIUM
ESTRADIOL
ESTRADIOL BENZOATE
ESTRADIOL CYPIONATE
ESTRADIOL DIPROPIONATE
ESTRADIOL VALERATE
ESTRAMUSTINE
ESTRIOL
ESTRONE
ESTROPIPATE
ETHACRYNIC ACID
ETHAMBUTOL HYDROCHLORIDE
ETHAVERINE HYDROCHLORIDE
ETHINYL ESTRADIOL
ETHIONAMIDE
ETHISTERONE
ETHOPROPAZINE HYDROCHLORIDE
ETHYL PARABEN
ETODOLAC
ETOPOSIDE
EUCALYPTOL
EUCATROPINE HYDROCHLORIDE
EUGENOL
EVANS BLUE
EXEMESTANE
EZETIMIBE
FAMCICLOVIR
FAMOTIDINE
FAMPRIDINE
FASUDIL HYDROCHLORIDE
FEBUXOSTAT
FENBENDAZOLE
FENBUFEN
FENDILINE HYDROCHLORIDE
FENOFIBRATE
FENOPROFEN
FENOTEROL HYDROBROMIDE
FENSPIRIDE HYDROCHLORIDE
FEXOFENADINE HYDROCHLORIDE
FIPEXIDE HYDROCHLORIDE
FIROCOXIB
FLOXURIDINE
FLUCONAZOLE
FLUDROCORTISONE ACETATE
FLUFENAMIC ACID
FLUINDAROL
FLUMEQUINE
FLUMETHASONE
FLUMETHAZONE PIVALATE
FLUNARIZINE HYDROCHLORIDE
FLUNISOLIDE
FLUNIXIN MEGLUMINE
FLUOCINOLONE ACETONIDE
FLUOCINONIDE
FLUOROMETHOLONE
FLUOROURACIL
FLUOXETINE
FLUPHENAZINE HYDROCHLORIDE
FLURANDRENOLIDE
FLURBIPROFEN
FLUROFAMIDE
FLUTAMIDE
FLUVASTATIN
FOLIC ACID
FOSCARNET SODIUM
FOSFOMYCIN CALCIUM
FTAXILIDE
FULVESTRANT
FURAZOLIDONE
FUROSEMIDE
FUSIDIC ACID
GABOXADOL HYDROCHLORIDE
GADOTERIDOL
GALANTHAMINE
GALLAMINE TRIETHIODIDE
GANCICLOVIR
GATIFLOXACIN
GEFITINIB
GEMFIBROZIL
GENTAMICIN SULFATE
GENTIAN VIOLET
GLIMEPIRIDE
GLUCONOLACTONE
GLUCOSAMINE HYDROCHLORIDE
GLUTAMINE (D)
GRAMICIDIN
GRANISETRON HYDROCHLORIDE
GRISEOFULVIN
GUAIFENESIN
GUANABENZ ACETATE
GUANETHIDINE SULFATE
HALAZONE
HALCINONIDE
HALOPERIDOL
HEPTAMINOL HYDROCHLORIDE
HETACILLIN POTASSIUM
HEXACHLOROPHENE
HEXYLRESORCINOL
HISTAMINE DIHYDROCHLORIDE
HOMATROPINE BROMIDE
HOMATROPINE METHYLBROMIDE
HOMOSALATE
HYCANTHONE
HYDRALAZINE HYDROCHLORIDE
HYDRASTINE (1R, 9S)
HYDROCHLOROTHIAZIDE
HYDROCORTISONE
HYDROCORTISONE ACETATE
HYDROCORTISONE BUTYRATE
HYDROCORTISONE HEMISUCCINATE
HYDROCORTISONE PHOSPHATE TRIETHYLAMINE
HYDROFLUMETHIAZIDE
HYDROQUINONE
HYDROXYAMPHETAMINE HYDROBROMIDE
HYDROXYCHLOROQUINE SULFATE
HYDROXYPROGESTERONE CAPROATE
HYDROXYTOLUIC ACID
HYDROXYUREA
HYDROXYZINE PAMOATE
HYOSCYAMINE
IBANDRONATE SODIUM
IBUPROFEN
IDOXURDINE
IDOXURIDINE
IMIPRAMINE HYDROCHLORIDE
IMIQUIMOD
INAMRINONE
INDAPAMIDE
INDOMETHACIN
INDOPROFEN
INOSITOL
IODIPAMIDE
IODIXANOL
IODOQUINOL
IOHEXOL
IOPANIC ACID
IOTHALAMIC ACID
IOVERSOL
IOXILAN
IPRATROPIUM BROMIDE
IRBESARTAN
ISONIAZID
ISOPROPAMIDE IODIDE
ISOPROTERENOL HYDROCHLORIDE
ISOSORBIDE DINITRATE
ISOSORBIDE MONONITRATE
ISOTRETINON
ISOXICAM
ISOXSUPRINE HYDROCHLORIDE
ITOPRIDE HYDROCHLORIDE
IVERMECTIN
KANAMYCIN A SULFATE
KETOCONAZOLE
KETOPROFEN
KETOROLAC TROMETHAMINE
KETOTIFEN FUMARATE
LABETALOL HYDROCHLORIDE
LACTULOSE
LAMIVUDINE
LANATOSIDE C
LANSOPRAZOLE
LEFLUNOMIDE
LETROZOLE
LEUCOVORIN CALCIUM
LEVAMISOLE HYDROCHLORIDE
LEVOCETIRIZINE DIHYDROCHLORIDE
LEVOFLOXACIN
LEVOMENTHOL
LEVONORDEFRIN
LEVONORGESTREL
LEVOSIMENDAN
LEVOTHYROXINE
LIDOCAINE HYDROCHLORIDE
LINCOMYCIN HYDROCHLORIDE
LINDANE
LINEZOLID
LIOTHYRONINE
LIOTHYRONINE (L- isomer) SODIUM
LISINOPRIL
LITHIUM CITRATE
LOBELINE HYDROCHLORIDE
LOFEXIDINE HYDROCHLORIDE
LOMEFLOXACIN HYDROCHLORIDE
LOMERIZINE HYDROCHLORIDE
LOMUSTINE
LORATADINE
LORNOXICAM
LOSARTAN
LOVASTATIN
LUMIRACOXIB
MAFENIDE HYDROCHLORIDE
MALATHION
MANGAFODIPIR TRISODIUM
MANIDIPINE HYDROCHLORIDE
MANNITOL
MAPROTILINE HYDROCHLORIDE
MEBENDAZOLE
MEBEVERINE HYDROCHLORIDE
MEBHYDROLIN NAPHTHALENESULFONATE
MECAMYLAMINE HYDROCHLORIDE
MECHLORETHAMINE
MECLIZINE HYDROCHLORIDE
MECLOCYCLINE SULFOSALICYLATE
MECLOFENAMATE SODIUM
MECLOFENOXATE HYDROCHLORIDE
MEDROXYPROGESTERONE ACETATE
MEDRYSONE
MEFENAMIC ACID
MEFEXAMIDE
MEFLOQUINE
MEGESTROL ACETATE
MEGLUMINE
MELOXICAM SODIUM
MELPERONE HYDROCHLORIDE
MELPHALAN
MEMANTINE HYDROCHLORIDE
MENADIONE
MEPARTRICIN
MEPENZOLATE BROMIDE
MEPHENESIN
MEPHENTERMINE SULFATE
MEPIVACAINE HYDROCHLORIDE
MEQUINOL
MERBROMIN
MERCAPTOPURINE
MEROPENEM
MESNA
MESO-ERYTHRITOL
MESTRANOL
METAPROTERENOL
METARAMINOL BITARTRATE
METAXALONE
METHACHOLINE CHLORIDE
METHACYCLINE HYDROCHLORIDE
METHAPYRILENE HYDROCHLORIDE
METHAZOLAMIDE
METHENAMINE
METHICILLIN SODIUM
METHIMAZOLE
METHOCARBAMOL
METHOTREXATE(+/-)
METHOXAMINE HYDROCHLORIDE
METHOXSALEN
METHSCOPOLAMINE BROMIDE
METHYCLOTHIAZIDE
METHYLBENZETHONIUM CHLORIDE
METHYLDOPA
METHYLERGONOVINE MALEATE
METHYLPREDNISOLONE
METHYLPREDNISOLONE SODIUM SUCCINATE
METHYLTHIOURACIL
METOCLOPRAMIDE HYDROCHLORIDE
METOPROLOL TARTRATE
METRONIDAZOLE
MEXILETINE HYDROCHLORIDE
MICONAZOLE NITRATE
MIDODRINE HYDROCHLORIDE
MIGLITOL
MILNACIPRAN HYDROCHLORIDE
MINAPRINE HYDROCHLORIDE
MINOCYCLINE HYDROCHLORIDE
MINOXIDIL
MITOMYCIN C
MITOTANE
MITOXANTRONE HYDROCHLORIDE
MOLSIDOMINE
MONENSIN SODIUM (monensin A is shown)
MONOBENZONE
MORANTEL CITRATE
MOXALACTAM DISODIUM
MOXIFLOXACIN HYDROCHLORIDE
MYCOPHENOLATE MOFETIL
MYCOPHENOLIC ACID
NABUMETONE
NADIDE
NADOLOL
NAFCILLIN SODIUM
NAFRONYL OXALATE
NALBUPHINE HYDROCHLORIDE
NALIDIXIC ACID
NALOXONE HYDROCHLORIDE
NALTREXONE HYDROCHLORIDE
NAPHAZOLINE HYDROCHLORIDE
NAPROXEN(+)
NAPROXOL
NATEGLINIDE
NEFAZODONE HYDROCHLORIDE
NEFOPAM
NELARABIN
NEOMYCIN SULFATE
NEOSTIGMINE BROMIDE
NEVIRAPINE
NIACIN
NICARDIPINE HYDROCHLORIDE
NICERGOLINE
NICLOSAMIDE
NICOTINYL ALCOHOL TARTRATE
NIFEDIPINE
NIFURSOL
NILUTAMIDE
NIMODIPINE
NITAZOXANIDE
NITRENDIPINE
NITROFURANTOIN
NITROFURAZONE
NITROMIDE
NOCODAZOLE
NOMIFENSINE MALEATE
NOREPINEPHRINE
NORETHINDRONE
NORETHINDRONE ACETATE
NORETHYNODREL
NORFLOXACIN
NORGESTREL
NORTRIPTYLINE
NOSCAPINE HYDROCHLORIDE
NOVOBIOCIN SODIUM
NYLIDRIN HYDROCHLORIDE
NYSTATIN
OCTOPAMINE HYDROCHLORIDE
OFLOXACIN
OLMESARTAN
OLMESARTAN MEDOXOMIL
OLSALAZINE SODIUM
OLSELTAMIVIR PHOSPHATE
OMEGA-3-ACID ESTERS (EPA shown)
ONDANSETRON
ORLISTAT
ORPHENADRINE CITRATE
OUABAIN
OXACILLIN SODIUM
OXALIPLATIN
OXCARBAZEPINE
OXETHAZAINE
OXIBENDAZOLE
OXIDOPAMINE HYDROCHLORIDE
OXOLINIC ACID
OXYBENZONE
OXYMETAZOLINE HYDROCHLORIDE
OXYPHENBUTAZONE
OXYPHENCYCLIMINE HYDROCHLORIDE
OXYQUINOLINE HEMISULFATE
OXYTETRACYCLINE
PACLITAXEL
PALIPERIDONE
PAPAVERINE HYDROCHLORIDE
PARACHLOROPHENOL
PARAROSANILINE PAMOATE
PARGYLINE HYDROCHLORIDE
PAROMOMYCIN SULFATE
PAROXETINE HYDROCHLORIDE
PEMETREXED
PENCICLOVIR
PENICILLAMINE
PENICILLIN G POTASSIUM
PENICILLIN V POTASSIUM
PENTOLINIUM TARTRATE
PENTOXIFYLLINE
PERGOLIDE MESYLATE
PERHEXILINE MALEATE
PERICIAZINE
PERINDOPRIL ERBUMINE
PERPHENAZINE
PHENACEMIDE
PHENAZOPYRIDINE HYDROCHLORIDE
PHENELZINE SULFATE
PHENINDIONE
PHENIRAMINE MALEATE
PHENOLPHTHALEIN
PHENTOLAMINE HYDROCHLORIDE
PHENYL AMINOSALICYLATE
PHENYLBUTAZONE
PHENYLEPHRINE HYDROCHLORIDE
PHENYLMERCURIC ACETATE
PHENYLPROPANOLAMINE HYDROCHLORIDE
PHENYTOIN SODIUM
PHTHALYLSULFATHIAZOLE
PHYSOSTIGMINE SALICYLATE
PILOCARPINE NITRATE
PIMOZIDE
PINDOLOL
PIOGLITAZONE HYDROCHLORIDE
PIPERACETAZINE
PIPERACILLIN SODIUM
PIPERAZINE
PIPERIDOLATE HYDROCHLORIDE
PIPERINE
PIPOBROMAN
PIRACETAM
PIRENPERONE
PIRENZEPINE HYDROCHLORIDE
PIROCTONE OLAMINE
PIROXICAM
PITAVASTATIN CALCIUM
PIZOTYLINE MALATE
POLYMYXIN B SULFATE
POTASSIUM p-AMINOBENZOATE
PRAMIPEXOLE DIHYDROCHLORIDE
PRAMOXINE HYDROCHLORIDE
PRASUGREL
PRAZIQUANTEL
PRAZOSIN HYDROCHLORIDE
PREDNICARBATE
PREDNISOLONE
PREDNISOLONE ACETATE
PREDNISONE
PRILOCAINE HYDROCHLORIDE
PRIMAQUINE DIPHOSPHATE
PRIMIDONE
PROADIFEN HYDROCHLORIDE
PROBENECID
PROBUCOL
PROCAINAMIDE HYDROCHLORIDE
PROCAINE HYDROCHLORIDE
PROCARBAZINE HYDROCHLORIDE
PROCHLORPERAZINE EDISYLATE
PROCYCLIDINE HYDROCHLORIDE
PROGESTERONE
PROGLUMIDE
PROMAZINE HYDROCHLORIDE
PROMETHAZINE HYDROCHLORIDE
PRONETALOL HYDROCHLORIDE
PROPAFENONE HYDROCHLORIDE
PROPANTHELINE BROMIDE
PROPIOLACTONE
PROPOFOL
PROPYLTHIOURACIL
PSEUDOEPHEDRINE HYDROCHLORIDE
PUROMYCIN HYDROCHLORIDE
PYRANTEL PAMOATE
PYRAZINAMIDE
PYRETHRINS
PYRIDOSTIGMINE BROMIDE
PYRILAMINE MALEATE
PYRIMETHAMINE
PYRITHIONE ZINC
PYRONARIDINE TETRAPHOSPHATE
PYRVINIUM PAMOATE
QUETIAPINE
QUINACRINE HYDROCHLORIDE
QUINAPRIL HYDROCHLORIDE
QUINESTROL
QUINETHAZONE
QUINIDINE GLUCONATE
QUININE SULFATE
QUIPAZINE MALEATE
RACEPHEDRINE HYDROCHLORIDE
RACTOPAMINE HYDROCHLORIDE
RAMIPRIL
RAMOPLANIN [A2 shown; 2mm]
RANITIDINE
RASAGILINE
RESERPINE
RESORCINOL
RESORCINOL MONOACETATE
RETAPAMULIN
RETINOL
RETINYL PALMITATE
RIBAVIRIN
RIFAMPIN
RITANSERIN
RITODRINE HYDROCHLORIDE
RITONAVIR
RIZATRIPTAN BENZOATE
ROFECOXIB
RONIDAZOLE
ROPINIROLE
ROSIGLITAZONE
ROSUVASTATIN CALCIUM
ROXARSONE
ROXATIDINE ACETATE HYDROCHLORIDE
ROXITHROMYCIN
RUFLOXACIN HYDROCHLORIDE
SACCHARIN
SALICIN
SALICYL ALCOHOL
SALICYLAMIDE
SALICYLANILIDE
SALINOMYCIN, SODIUM
SALSALATE
SANGUINARINE SULFATE
SCOPOLAMINE HYDROBROMIDE
SELAMECTIN
SEMUSTINE
SENNOSIDE A
SERATRODAST
SERTRALINE HYDROCHLORIDE
SEVOFLURANE
SIBUTRAMINE HYDROCHLORIDE
SILDENAFIL CITRATE
SIMVASTATIN
SIROLIMUS
SISOMICIN SULFATE
SODIUM DEHYDROCHOLATE
SODIUM NITROPRUSSIDE
SODIUM OXYBATE
SODIUM PHENYLACETATE
SODIUM PHENYLBUTYRATE
SODIUM SALICYLATE
SPARFLOXACIN
SPARTEINE SULFATE
SPECTINOMYCIN HYDROCHLORIDE
SPIPERONE
SPIRAMYCIN
SPIRAPRIL HYDROCHLORIDE
SPIRONOLACTONE
STAVUDINE
STREPTOMYCIN SULFATE
STREPTOZOSIN
SUCCINYLSULFATHIAZOLE
SULBACTAM
SULCONAZOLE NITRATE
SULFABENZAMIDE
SULFACETAMIDE
SULFACHLORPYRIDAZINE
SULFADIAZINE
SULFADIMETHOXINE
SULFAMERAZINE
SULFAMETER
SULFAMETHAZINE
SULFAMETHIZOLE
SULFAMETHOXAZOLE
SULFAMETHOXYPYRIDAZINE
SULFAMONOMETHOXINE
SULFANILATE ZINC
SULFANITRAN
SULFAPYRIDINE
SULFAQUINOXALINE SODIUM
SULFASALAZINE
SULFATHIAZOLE
SULFINPYRAZONE
SULFISOXAZOLE
SULINDAC
SULMAZOLE
SULOCTIDIL
SULPIRIDE
SUPROFEN
SURAMIN
TACROLIMUS
TAMOXIFEN CITRATE
TANDUTINIB
TANNIC ACID
TAZOBACTAM
TEGASEROD MALEATE
TELMISARTAN
TEMEFOS
TEMOZOLAMIDE
TENIPOSIDE
TENOXICAM
TERBUTALINE HEMISULFATE
TERCONAZOLE
TERFENADINE
TESTOSTERONE
TESTOSTERONE PROPIONATE
TETRACAINE HYDROCHLORIDE
TETRACYCLINE HYDROCHLORIDE
TETRAHYDROZOLINE HYDROCHLORIDE
TETROQUINONE
THALIDOMIDE
THEOBROMINE
THEOPHYLLINE
THIABENDAZOLE
THIAMPHENICOL
THIMEROSAL
THIOGUANINE
THIORIDAZINE HYDROCHLORIDE
THIOSTREPTON
THIOTEPA
THIOTHIXENE
THIRAM
THONZONIUM BROMIDE
THONZYLAMINE HYDROCHLORIDE
TIAPRIDE HYDROCHLORIDE
TIBOLONE
TIGECYCLINE
TILARGININE HYDROCHLORIDE
TILETAMINE HYDROCHLORIDE
TILMICOSIN
TIMOLOL MALEATE
TINIDAZOLE
TOBRAMYCIN
TODRALAZINE HYDROCHLORIDE
TOLAZAMIDE
TOLAZOLINE HYDROCHLORIDE
TOLBUTAMIDE
TOLMETIN SODIUM
TOLNAFTATE
TOLPERISONE HYDROCHLORIDE
TOSYLCHLORAMIDE SODIUM
TRANEXAMIC ACID
TRANYLCYPROMINE SULFATE
TRAZODONE HYDROCHLORIDE
TRETINOIN
TRIACETIN
TRIAMCINOLONE
TRIAMCINOLONE ACETONIDE
TRIAMCINOLONE DIACETATE
TRIAMTERENE
TRICHLORMETHIAZIDE
TRIFLUOPERAZINE HYDROCHLORIDE
TRIFLUPROMAZINE HYDROCHLORIDE
TRIFLURIDINE
TRIHEXYPHENIDYL HYDROCHLORIDE
TRILOSTANE
TRIMEPRAZINE TARTRATE
TRIMETHOBENZAMIDE HYDROCHLORIDE
TRIMETHOPRIM
TRIMETOZINE
TRIMIPRAMINE MALEATE
TRIOXSALEN
TRIPELENNAMINE CITRATE
TRIPROLIDINE HYDROCHLORIDE
TRISODIUM ETHYLENEDIAMINE TETRACETATE
TROLEANDOMYCIN
TROPICAMIDE
TROPISETRON HYDROCHLORIDE
TRYPTOPHAN
TUAMINOHEPTANE SULFATE
TUBOCURARINE CHLORIDE
TYROTHRICIN
URACIL
URAPIDIL HYDROCHLORIDE
UREA
URETHANE
URSODIOL
VALDECOXIB
VALGANCICLOVIR HYDROCHLORIDE
VALPROATE SODIUM
VALSARTAN
VANCOMYCIN HYDROCHLORIDE
VENLAFAXINE
VIDARABINE
VINBLASTINE SULFATE
VINORELBINE
VINPOCETINE
VIOMYCIN SULFATE
VORICONAZOLE
VORINOSTAT
WARFARIN
XYLAZINE
XYLOMETAZOLINE HYDROCHLORIDE
YOHIMBINE HYDROCHLORIDE
ZALCITABINE
ZAPRINAST
ZIDOVUDINE [AZT]
ZIPRASIDONE MESYLATE
ZOMEPIRAC SODIUM
ZOPICLONE

### Data analysis

Data are presented as the mean and SEM, unless stated otherwise. Pairwise statistical significance was determined with a Student’s two-tailed unpaired *t* test, ANOVA, or Mann–Whitney rank sum test, as appropriate, unless stated otherwise. Results were considered significant at *p* < 0.05, unless otherwise indicated.

## Results

### A first-stage behavioral screen for antiepileptic activity

**Figure 2.**
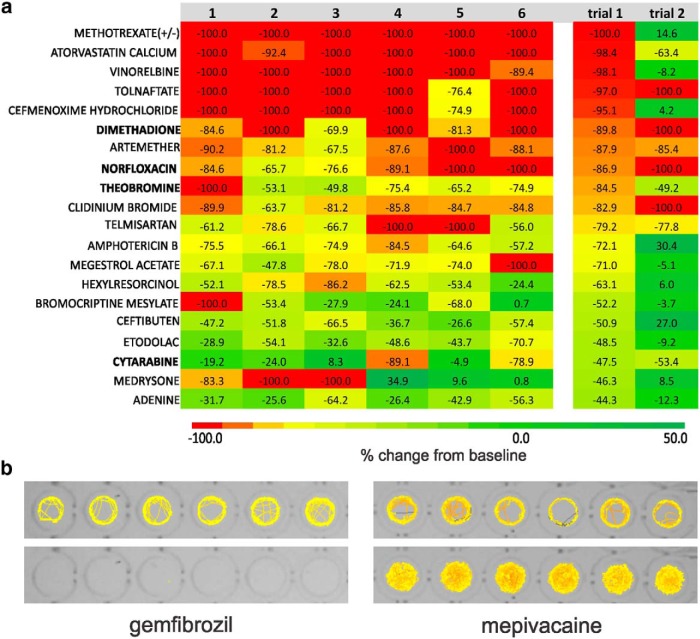
Positive hits identified in the locomotion assay. ***a***, Heat map showing the results of individual zebrafish trials (1-6) for compounds tested at a concentration of 100 µm in the locomotion-tracking assay. Raw data values for individual fish are shown within the color-coded boxes for one sample trial. Mean velocity data are shown at right for “trial 1” and “trial 2”; six fish per trial. Note: only drugs highlighted in bold type were classified as positive nontoxic hits in two independent trials and moved on to further testing. ***b***, Representative raw locomotion data plots for six individual *scn1Lab* mutant larvae at baseline (top) and following the addition of a compound resulting in fatality (bottom, gemfibrozil) or hyperactivity (bottom, mepivacaine). Movement is color coded, with low-velocity movements shown in yellow, and high velocity movements shown in red.

Locomotion tracking is a reliable and rapid strategy with which to monitor behavioral seizures in freely swimming larval zebrafish (Baraban et al., 2005, 2013; Winter et al., 2008). In these locomotion plots, high-velocity movements of ≥20 mm/s correspond to paroxysmal whole-body convulsions, referred to as Stage III, and are consistently observed in unprovoked *scn1Lab* mutant larvae but not in age-matched wild-type siblings. Using automated locomotion tracking, we performed a phenotype-based screen to identify compounds that significantly reduce mutant swim behavior to levels associated with Stage 0 or Stage I (e.g., activity equivalent to that seen in normal untreated WT zebrafish). In a 96-well format, we tracked mutant swim activity at baseline, and then again after addition of a test compound (100 µm); each compound was tested on six individual mutant larvae (Fig. 1*a*), and larvae were sorted based on pigmentation differences (Fig. 1*b*). Mutant swim activity between two consecutive recording epochs in embryo media is tracked on every plate as an internal control. A box plot showing the change in swim velocity in untreated mutants is shown in Figure 1*c* (*n* = 112) and defined as the control. Based on an SD of 21.8 for these data, we set the detection threshold as any compound that inhibits movement (measured as a change in mean velocity) by >2 SDs (or ≥44%). This approach was previously validated using standard antiepileptic drugs in this model (Baraban et al., 2013). Next, we screened a repurposed library in which all compounds have reached the clinical evaluation stage (PHARMAKON 1600 Collection; http://www.msdiscovery.com/pharma.html). Among the 1012 compounds screened (Fig. 1*d*) only 20 (or 1.97%) were found to significantly inhibit spontaneous seizure behavior in *scn1Lab* mutants. All 20 compounds were subsequently retested in a separate clutch of *scn1Lab* mutants at a concentration of 100 µm (Fig. 2*a*, trial 2; *N* = 6 fish/compound). A total of 154 compounds were classified as “toxic” (Table 2); 55 compounds were classified as “hyperexcitable” (Table 3). Representative locomotion tracking raw data plots for gemfibrozil, a toxic nonsteroid nuclear receptor ligand, and mepivacaine, a hyperexcitable proconvulsant anesthetic, are shown in Figure 2*b*.

**Table 2:** List of compounds exhibiting toxicity.

ABACAVIR SULFATE
ACIPIMOX
ADENOSINE PHOSPHATE
ALAPROCLATE
AMLEXANOX
AMOROLFINE HYDROCHLORIDE
ANTAZOLINE PHOSPHATE
ARTEMETHER
ASCORBIC ACID
ATORVASTATIN CALCIUM
AUROTHIOGLUCOSE
AZELAIC ACID
BENORILATE
BENZONATATE
BETAINE HYDROCHLORIDE
BETAMIPRON
BROMHEXINE HYDROCHLORIDE
BUDESONIDE
BUPIVACAINE HYDROCHLORIDE
BUSULFAN
BUTOCONAZOLE
CAPSAICIN
CARPROFEN
CEFORANIDE
CEFOTAXIME SODIUM
CEFOXITIN SODIUM
CEPHALEXIN
CHLORAMBUCIL
CHLORAMPHENICOL HEMISUCCINATE
CHLOROGUANIDE HYDROCHLORIDE
CHLORPHENIRAMINE (S) MALEATE
CINCHOPHEN
CINNARAZINE
CINTRIAMIDE
CIPROFLOXACIN
CLIDINIUM BROMIDE
CLOZAPINE
COLISTIMETHATE SODIUM
CRYOFLURANE
CYCLOPHOSPHAMIDE HYDRATE
CYCLOTHIAZIDE
CYPERMETHRIN
DAUNORUBICIN
DECIMEMIDE
DEXTROMETHORPHAN HYDROBROMIDE
DICHLOROPHENE
DIETHYLCARBAMAZINE CITRATE
DIOXYBENZONE
DIRITHROMYCIN
DISOPYRAMIDE PHOSPHATE
DISULFIRAM
ECONAZOLE NITRATE
EDETATE DISODIUM
EMETINE
ENALAPRILAT
ERYTHROMYCIN
ETHINYL ESTRADIOL
ETHIONAMIDE
ETHOPROPAZINE HYDROCHLORIDE
ETHYL PARABEN
EUGENOL
FIPEXIDE HYDROCHLORIDE
FLUMETHASONE
FLUNISOLIDE
FLUVASTATIN
GEMFIBROZIL
GENTAMICIN SULFATE
GLUCONOLACTONE
HALAZONE
HALCINONIDE
HETACILLIN POTASSIUM
HEXACHLOROPHENE
HOMATROPINE METHYLBROMIDE
HYDRASTINE (1R, 9S)
HYDROXYAMPHETAMINE HYDROBROMIDE
HYDROXYCHLOROQUINE SULFATE
IODIXANOL
IOHEXOL
IRBESARTAN
LEVOSIMENDAN
LISINOPRIL
LOMERIZINE HYDROCHLORIDE
MANGAFODIPIR TRISODIUM
MECLOFENOXATE HYDROCHLORIDE
MESTRANOL
METHACHOLINE CHLORIDE
METHYLERGONOVINE MALEATE
METRONIDAZOLE
MIGLITOL
MONENSIN SODIUM (monensin A is shown)
MONOBENZONE
MOXALACTAM DISODIUM
NADOLOL
NALBUPHINE HYDROCHLORIDE
NALTREXONE HYDROCHLORIDE
NAPHAZOLINE HYDROCHLORIDE
NAPROXEN(+)
NEOMYCIN SULFATE
NIFEDIPINE
NITAZOXANIDE
NITROMIDE
NORETHINDRONE
OLMESARTAN MEDOXOMIL
OXYMETAZOLINE HYDROCHLORIDE
PARACHLOROPHENOL
PAROMOMYCIN SULFATE
PERHEXILINE MALEATE
PHENTOLAMINE HYDROCHLORIDE
PHENYLBUTAZONE
PHENYLMERCURIC ACETATE
PHYSOSTIGMINE SALICYLATE
PIMOZIDE
PIPERACILLIN SODIUM
PIPERAZINE
PIRACETAM
PIRENZEPINE HYDROCHLORIDE
PIROCTONE OLAMINE
PITAVASTATIN CALCIUM
PRIMAQUINE DIPHOSPHATE
PROBENECID
PROCARBAZINE HYDROCHLORIDE
PROGLUMIDE
PROMETHAZINE HYDROCHLORIDE
PUROMYCIN HYDROCHLORIDE
QUININE SULFATE
RETINYL PALMITATE
RIFAMPIN
RITONAVIR
ROFECOXIB
RUFLOXACIN HYDROCHLORIDE
SACCHARIN
SALICIN
SENNOSIDE A
STAVUDINE
STREPTOMYCIN SULFATE
SULFADIAZINE
SULINDAC
SULOCTIDIL
TANNIC ACID
TELMISARTAN
TENOXICAM
THEOPHYLLINE
TILETAMINE HYDROCHLORIDE
TILMICOSIN
TIMOLOL MALEATE
TOLBUTAMIDE
TOLNAFTATE
TRAZODONE HYDROCHLORIDE
TRETINOIN
TRIFLUPROMAZINE HYDROCHLORIDE
TROPISETRON HYDROCHLORIDE
VALDECOXIB
VORINOSTAT
ZALCITABINE

**Table 3:** List of compounds exhibiting hyperexcitable or proconvulsant activity.

ADENOSINE PHOSPHATE
ALBUTEROL (+/-)
ALEXIDINE HYDROCHLORIDE
AMANTADINE HYDROCHLORIDE
AMINOHIPPURIC ACID
AMINOLEVULINIC ACID HYDROCHLORIDE
AUROTHIOGLUCOSE
AZACITIDINE
BENZOYL PEROXIDE
BETAZOLE HYDROCHLORIDE
BROMHEXINE HYDROCHLORIDE
BUSULFAN
CEFSULODIN SODIUM
CEFUROXIME AXETIL
CHLOROGUANIDE HYDROCHLORIDE
CYSTEAMINE HYDROCHLORIDE
ECAMSULE TRIETHANOLAMINE
ECONAZOLE NITRATE
EDOXUDINE
ENROFLOXACIN
ESTRADIOL CYPIONATE
ETHINYL ESTRADIOL
ETHOPROPAZINE HYDROCHLORIDE
ETOPOSIDE
FASUDIL HYDROCHLORIDE
FEBUXOSTAT
FLUMETHASONE
FLUOROMETHOLONE
FURAZOLIDONE
GANCICLOVIR
GLUCONOLACTONE
GRANISETRON HYDROCHLORIDE
HALAZONE
HEXACHLOROPHENE
IODIPAMIDE
LABETALOL HYDROCHLORIDE
MEPIVACAINE HYDROCHLORIDE
MITOXANTRONE HYDROCHLORIDE
MORANTEL CITRATE
NOCODAZOLE
OFLOXACIN
PENTOLINIUM TARTRATE
PERINDOPRIL ERBUMINE
PIOGLITAZONE HYDROCHLORIDE
PRAMIPEXOLE DIHYDROCHLORIDE
PROGLUMIDE
RIFAMPIN
SERATRODAST
SERTRALINE HYDROCHLORIDE
SIBUTRAMINE HYDROCHLORIDE
SUCCINYLSULFATHIAZOLE
TACROLIMUS
TETROQUINONE
TIMOLOL MALEATE
URACIL

### A second-stage electrophysiology assay for antiepileptic activity

**Figure 3.**
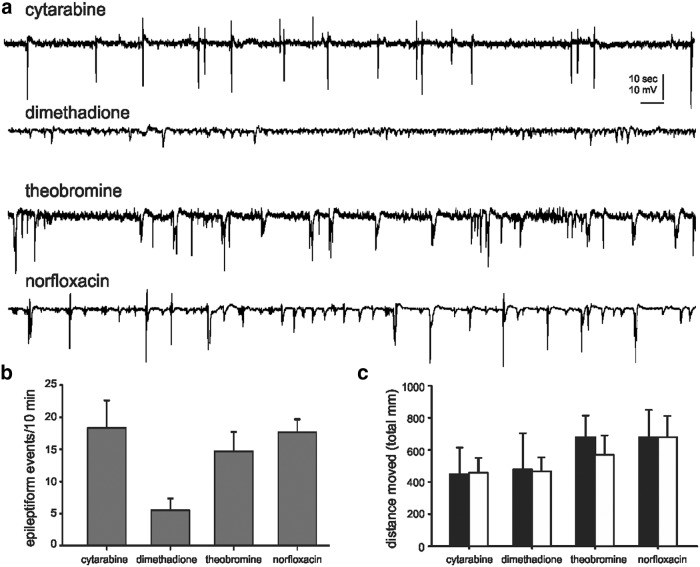
Electrophysiology assay to identify drugs that rescue the *scn1Lab* mutant epilepsy phenotype. ***a***, Representative field electrode recording epochs (5 min in duration) are shown for the “positive” compounds identified in the locomotion assay. All recordings were obtained with an electrode placed in the forebrain of agar-immobilized *scn1Lab* larvae that was previously tested in the locomotion assay. A suppression of epileptiform electrographic discharge activity was noted in mutants exposed to dimethadione. ***b***, Bar plot showing the mean number of epileptiform events in a 10 min recording epoch for *scn1Lab* larvae exposed to cytarabine (*N* = 6), dimethadione (*N* = 6), theobromine (*N* = 6), and norfloxacin (*N* = 6). The mean ± SEM is shown. The fish shown were tested in the locomotion assay first. ***c***, Bar plot showing the total distance traveled before (black bars) and after (white bars) exposure to a test compound; 10 min recording epoch and six fish per drug. The mean ± SEM is shown.

Extracellular recording electrodes are a reliable, reproducible, and sensitive approach to monitor electroencephalographic activity in agar-immobilized larval zebrafish (Baraban et al., 2005; Baraban, 2013). Field electrodes offer high a signal-to-noise ratio and can be placed, using direct visualization in transparent larvae, into specific CNS structures (i.e., telencephalon or optic tectum). Using a local field electrode, we can efficiently monitor the occurrence of electrographic seizure events in the same zebrafish that were previously tested in the locomotion assay. Based on a positive nontoxic result in two independent locomotion assays, four drugs moved on to electrophysiology testing at concentrations between 500 µm and 1 mm (Fig. 3). Consistent with a “false-positive” classification, spontaneous epileptiform discharge activity was observed for three of these drugs: norfloxacin, theobromine, and cytarabine. Dimethadione, previously shown to inhibit spontaneous epileptiform discharges in thalamocortical slices at concentrations between 1 and 10 mm (Zhang et al., 1996), suppressed burst discharge activity in *scn1Lab* mutant larvae (Fig. 3*a*,*b*). To identify whether any of these four compounds exert nonspecific effects on behavior, they were also tested on freely swimming WT zebrafish larvae (5 dpf) at a concentration of 500 µm. Comparing the total distance moved during a 10 min recording epoch before, and after, the application of a test compound failed to reveal any significant changes in locomotor activity (Fig. 3*c*).

### Assessment of huperzine A and fenfluramine for antiepileptic activity

**Figure 4.**
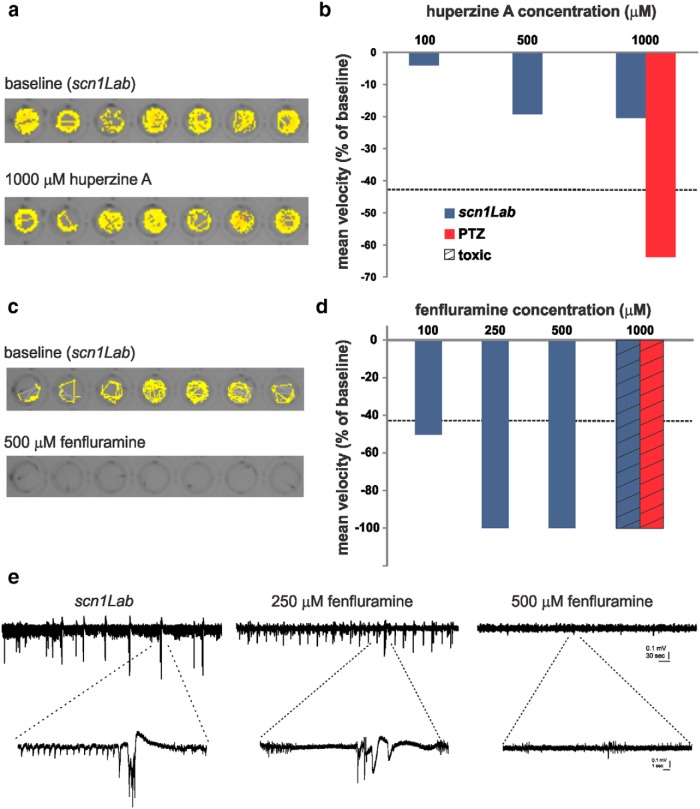
Evaluation of putative antiepileptic drugs in *scn1Lab* mutants. ***a***, Locomotion tracking plots for *scn1Lab* zebrafish at baseline and following huperzine A administration. Total movement is shown for a 10 min recording epoch. ***b***, Plot showing the change in mean velocity for three different huperzine A concentrations (blue bars). Each bar is the mean change for six fish. The threshold for a positive hit is shown as a dashed line. WT fish exposed to PTZ and huperzine A are shown in red (*N* = 7). ***c***, ***d***, Same for fenfluramine. Note that 1 mm fenfluramine was toxic, as indicated. ***e***, Representative field recordings from *scn1Lab* mutant larvae at 5 dpf. Electrographic activity is shown for a 5 min recording epoch (top traces); high-resolution traces are shown below, as indicated. Note that abnormal burst discharge activity persists in *scn1Lab* mutants exposed to 250 µm fenfluramine. The fish shown were tested in the locomotion assay first.

Next, we tested two additional compounds that were not in our drug library, but have recently been described as potential antiepileptic treatments for DS. Huperzine A, a small-molecule alkaloid isolated from Chinese club moss with NMDA-type receptor blocking and anticholinesterase activity, has purported antiepileptic actions against NMDA- or soman-induced seizures (Tonduli et al., 2001; Coleman et al., 2008). In the locomotion assay, huperzine A failed to significantly alter *scn1Lab* seizure behavior at any concentration tested (Fig. 4*a*,*b*). In contrast, huperzine A was effective at 1 mm in the acute pentylenetetrazole (PTZ) assay (Fig. 4*b*). Fenfluramine is an amphetamine-like compound that has been reported to successfully reduce seizure occurrence in children with DS as a low-dose add-on therapy (Ceulemans et al., 2012). In the locomotion assay, fenfluramine significantly reduced mutant mean swim velocity at concentrations between 100 and 500 μm (Fig. 4*c*,*d*); 1 mm fenfluramine was toxic in the *scn1Lab* and PTZ assays (Fig. 4*d*). The fenfluramine-treated *scn1Lab* mutant exhibited a suppression of spontaneous electrographic seizure discharge to levels similar to controls at 500 μm, but only a partial reduction in electrographic activity at 250 μm (Fig. 4*e*).

## Discussion

Zebrafish and humans share extensive genomic similarity. With regard to disease, 84% of genes known to be associated with disease states in humans have a zebrafish homolog (Howe et al., 2013). This genetic similarity and the characteristic of zebrafish larvae to exhibit quantifiable seizure behaviors or electrographic seizure discharge that is fundamentally similar to that recorded in humans (Jirsa et al., 2014) make this an ideal system for drug discovery. Behavioral assays customized for automated evaluation of locomotion (Winter et al., 2008; Creton, 2009; Baxendale et al., 2012; Baraban et al., 2013; Raftery et al., 2014) make moderate-to-high-throughput phenotype-based drug screening in zebrafish possible. Using this approach and a zebrafish *scn1* mutant (Baraban et al., 2013), we successfully identified antiepileptic compounds. Here we report results from screening ∼1000 compounds from a repurposed drug library and present data that will be periodically updated on-line using this open-access publishing mechanism.

As a model system, the *scn1Lab* mutant zebrafish has many advantages. First, in contrast to transient and variable knockdown of gene expression using antisense morpholino oligonucleotides (Teng et al., 2010; Finckbeiner et al., 2011; Mahmood et al., 2013), *scn1Lab* mutants carry a stable and heritable amino acid substitution at position 1208 in the third domain of *SCN1A* that shares 76% homology with humans (Schoonheim et al., 2010; Baraban et al., 2013). Mutations in this channel are one of the primary genetic causes underlying DS (Claes et al., 2003; Escayg and Goldin, 2010; De Jonghe, 2011; Saitoh et al., 2012). As zebrafish possess two *scn1* genes (Novak et al., 2006), homozygous mutants for *scn1Lab* are comparable to the haploinsufficient clinical condition, and there is no variability from larvae to larvae, or clutch to clutch, with respect to gene inactivation, as is commonly observed with morpholino injections (Kok et al., 2015). Although crosses of heterozygotes produce only one-quarter homozygous *scn1Lab* mutants per mating, there are virtually no limitations on maintaining a large colony of healthy, adult breeders for these types of large-scale screens. Second, it is possible to observe and monitor seizure-like behavior consisting of rapid movements and whole-body convulsions in freely swimming *scn1Lab* mutants as early as 4 dpf that persist for the life of the larvae (∼12 dpf). These behaviors are comparable to those observed with exposure to a common convulsant agent (PTZ) and classified as Stage III (Baraban et al., 2005). In addition, clear evidence for epileptiform discharge generated in the CNS of immobilized *scn1Lab* mutant larvae has been obtained at ages between 4 and 8 dpf (Baraban et al., 2013). Both zebrafish measures of seizure activity are sensitive to inhibition by AEDs commonly prescribed to children with DS (e.g., valproate, benzodiazepines, and stiripentol), but are resistant to many antiepileptic compounds (e.g., phenytoin, carbamazepine, ethosuximide, decimemide, tiletamine, primidone, phenacemide, and vigabatrin). Pharmacoresistance is defined as the inability to control seizure activity with at least two different AEDs (Berg, 2009), and, with demonstrated resistance to eight AEDs, our model clearly fits this definition. This level of model validation has not been possible with morpholinos probably owing to the high degree of variability, or off-target effects, associated with this technique (Kok et al., 2015).

Our screening results highlight the stringency of our approach with a positive hit rate of only 1.97% on the first-stage locomotion assay, and successful identification of 1 compound (of 1012 compounds) with known antiepileptic activity (i.e., dimethadione, a T-type channel antagonist). In additional testing, we confirmed an antiepileptic action for fenfluramine (serotonin uptake inhibitor). Similar to ethosuximide, a reduction in regenerative burst discharges associated with neuronal T-type calcium currents could be the underlying mechanism for dimethadione in DS mutants; however, it is worth noting that T-type channel blockers ethosuximide and flunarizine were not similarly effective (Baraban et al. 2013; this article), and that dimethadione can cause arrhythmia owing to blockade of cardiac human ether-a-go-go-related gene potassium channels (Azarbayjani and Danielsson, 2002; Danielsson et al., 2007). Modulation of serotonin [5-hydroxytryptamine (5-HT)] signaling by blocking uptake or increasing release from neurons by acting as substrates for 5-HT transporter (sertraline) proteins (Fuller et al., 1988; Gobbi and Mennini, 1999; Baumann et al., 2000; Rothman et al., 2010) may be the mechanism of action for fenfluramine in patients with DS, though a detailed analysis of precisely how fenfluramine modulates excitability via this signaling pathway has not been performed. Nonetheless, both drugs probably exert a direct effect on network excitability (at neuronal or synaptic levels, respectively) to suppress electrographic discharge and the associated high-velocity seizure behavior seen in *scn1Lab* mutants, and may be potential targets for clinical use. In contrast, three other drugs identified in the primary locomotion assay were not effective in suppressing electrical events and were designated as false positives. This is not altogether surprising given that the xanthine alkaloid (theobromine), chemotherapeutic (cytarabine), and antibiotic (norfloxacin) mechanisms for these compounds would not be consistent with seizure inhibition. Moreover, the variability inherent in behavioral experiments performed on different zebrafish larvae, in different microplates, and on different days may contribute to these false-positive designations in locomotion assays, and is evident in the range of mean velocity values seen during tracking episodes from control studies (Fig. 1*c*) or in the failure of many of the initial 20 lead compounds to be confirmed on subsequent retesting (see Fig. 2*a*). This is a limitation of locomotion-based screening assays and is another reason why a secondary electrophysiology assay on the same zebrafish is a critical advantage of our approach.

An additional advantage of *in vivo* screening with zebrafish larvae is the simultaneous identification of compounds resulting in toxicity. Zebrafish-based anticonvulsant drug-screening assays based primarily on *in situ* hybridization detection of early gene expression at 2 dpf (Baxendale et al., 2012) do not routinely monitor spontaneous swim behavior, heart rate, or response to external stimuli. Lacking these real-time measures of toxicity, compounds observed to induce fatality in a freely swimming *scn1Lab*-based behavioral assay (e.g., gemfibrozil, suloctidil, pimozide, or dioxybenzone) were mistakenly classified as seizure-suppressing compounds in the PTZ-based c-Fos *in situ* hybridization assay. Indeed, 41% of the “anticonvulsant” compounds positively identified at 2 dpf in Baxendale et al. (2012) were toxic, proconvulsant, or simply not effective in *scn1Lab* mutant assays at 5-6 dpf. Similarly, it is critical to monitor blood flow and heart activity even in the agar-immobilized electrophysiology assay as compounds effective in suppressing electrical activity can also be toxic. These discrepancies highlight the potential problems associated with zebrafish drug-screening strategies that do not encompass multiple readouts and suggest the need for a note of caution when comparing screening results from different laboratory groups. While any lead compound identified in a zebrafish-based screening assay will, ultimately, need to be independently replicated and/or validated in additional mammalian model systems, the ability to rapidly identify such compounds, while simultaneously identifying potential negative side effects, makes genetically modified zebrafish a unique resource for drug discovery in an age of personalized medicine.

## Synthesis

The decision was a result of the Reviewing Editor Elizabeth Powell and the peer reviewers coming together and discussing their recommendations until a consensus was reached. A fact-based synthesis statement explaining their decision and outlining what is needed to prepare a revision is listed below. The following reviewers agreed to reveal their identity: Karen Wilcox, Rosane Da Silva

Synthesis

The manuscript describes a high throughput study in which a zebrafish model of Dravet syndrome is uses to screen more than 1000 compounds in the search for new drugs for treatment in human patients. The reviewers stated strong enthusiasm for the work and the potential for advancing the field of human epilepsy and new therapeutic compounds. The format of the eNeuro journal is appropriate, as the data sets can be later updated to include new findings. Publishing all the data, the effective and ineffective or harmful compounds, is an important resource for the field.

Some major and minor issues were raised by the reviewers. These issues should be addressed prior to the manuscript being considered for publication. The reviewers' comments follow.

Reviewer #1

This is a well described, effective screen that has identified a number of potential anti-seizure drugs (ASDs) that may be efficacious for the treatment of Dravet's syndrome and other types of epilepsy. This approach should streamline ASD discovery and provide the field with useful positive and perhaps more importantly, negative results (so as to not waste time and effort on ineffective compounds). There are some issues that should be addressed.

Major concern:

There seems to be considerable variability in trial to trial effectiveness of compounds (e.g. Figure 3, with a stated 4/20 compounds showing comparable reductions in movement velocity from trial 1 to trial 2). The authors are to be commended for performing independent validation of each trial. However, the variability is not discussed in the context of being a limitation of this approach. Also, the threshold for considering whether a compound is indeed a positive hit is not well described. For example it is not clear why Theobromine was considered a hit in Fig 3, but Tolnaftate and Artemether was not. These issues should be discussed.

Minor Concerns:

In Figure 4, it would be helpful to provide baseline recording segments prior to the administration of compounds, especially any that prove efficacious (e.g. dimethadione).

In the discussion, towards the end of the second paragraph, the author states "...and successful identification of two compounds.....and fenfluramine". However, in the results section, it was mentioned that fenfluramine was not part of the original screen but was tested as a consequence of cited literature. In the discussion, the way that sentence is phrased, it sounds like fenfluramine was found in the screen of the library.

Figure 2 is somewhat confusing. At what point is it determined that velocity and distance are not correlated. It is unclear why the drugs selected were plotted, as none were viewed as significant hits. Also, as a positive hit needed to demonstrate >44% reduction in velocity, it is not clear why estradiol was not considered a positive hit? Were the 5 drugs in part B part of the library?

Reviewer #2

This manuscript describes a large-scale drug screening to identify antiepileptic drugs based in locomotor activity and electroencephalography registration using zebrafish. The study is well conducted and its publication will be a huge contribution to the use of zebrafish as a model to investigate antiepileptic properties and a contribution to the study of refractory epilepsy. While I am enthusiastic about the results of this manuscript, my enthusiasm became a little bit vanished while reading the text. In this way, I strongly recommend a careful revision of the text, especially in relation to the points mentioned below:

Major points:

1. While the methodological approach is one of the main contributions of the study the authors do not discuss the results on the perspectives of the disease. The authors should contextualize the results in the end of the abstract and through the discussion session in order to show how the drugs identified could contribute to the study of the Dravet Syndrome.

2. Also, the authors do not discuss the findings on the mechanistic level of the drugs identified with antiepileptic properties.

3. On the introduction, the authors should enrich this session with data from the Dravet syndrome, treatments available...

4. There is an important problem in the distribution of the text through the sessions. There are elements of material and methods, results and discussion sessions on the introduction. There is lot of comments in the material and methods results sessions that should be avoided or transferred to the discussion session. The material and methods should not include results (e.g: last sentence of Drugs subtitle). The result session is exclusively to the results reached from this specific study, please delete comments, justifications,...

5. The material and methods session is very poorly written and there are several methodological information through the others sessions. See examples below:

5.1 What is the guideline used to base the animal care?

5.2 What conditions the animals were maintained (temperature, pH, conductivity, light/dark conditions, density, tanks dimension, 96 wells plate ...)?

5.3 The age of larvae during the seizure monitoring experiments is lacking (this information appears just in the legend of the figure 1)

5.4 How the heart beating and blood flow were registered?

5.5 How the animals were exposed to the drugs? How long was the exposition? Is the 15-30 minutes of equilibration period the time of exposure?

5.6 Was the final DMSO concentration in the electrophysiology experiments the same of locomotor experiments?

5.7 The concept of "% changes in the mean velocity" should be stated in the material and methods and not in the result session.

5.8 The criteria to define a drug as "toxic" should be stated in the material and methods and not in the result session.

5.9 Please, explain the criteria to consider a drug as an anticonvulsant or a proconvulsant at the material and methods and not at the results session.

5.10 Please, was the mortality registered?

6. In the results session, the authors mentioned that the animals were sorted based on the pigmentation. What were the parameters considered? Please, at least mention that in the material and methods session.

7. Also in the results session, the authors should show the results of heart beat and blood flow, since at the discussion they mentioned that as an important data to avoid misunderstandings on the interpretation of data. This would help to differentiate the animals from gemfibrozil (Figure 3b) from fenfluramine (figure 5c), since the first was fatal and the second not.

8. The figures should include the data from WT animals and not just the mutant at the free-drug condition (locomotion and electrophysiology data).

9. At figure 1d, it is hard to identify the "1.97%" of compounds at the figure 1. The figure is a little bit crowded.

10. At the figure 4, why just 3 animals were used to the electrophysiology assay (cytarabine, theobromine and norfloxacin) since the locomotor assay was performed with 6 animals?

11. Why the statistic of the results is not shown in the figures? For example, it is hard to identify how the authors differentiated the groups at the figure 2.

12. Why the raw data were show just to the first trial?

Minor points:

1. Please, avoid the words very little, higher, increased swimming, when the basal data is not available. If possible, show the real data.

2. There are no figures 1e or 3c. Please, correct to 1d and 3b.

3. Please, organize the legend of figure 5, there is some repetition.

4. Why sometimes the total number of drugs is 1012, sometimes 1013, 1014..?
